# Systems Pharmacology Approach to Investigate the Mechanism of Kai-Xin-San in Alzheimer’s Disease

**DOI:** 10.3389/fphar.2020.00381

**Published:** 2020-04-03

**Authors:** Yunxia Luo, Dongli Li, Yanfang Liao, Chuipu Cai, Qihui Wu, Hanzhong Ke, Xinning Liu, Huilin Li, Honghai Hong, Yumin Xu, Qi Wang, Jiansong Fang, Shuhuan Fang

**Affiliations:** ^1^Science and Technology Innovation Center, Guangzhou University of Chinese Medicine, Guangzhou, China; ^2^Department of Endocrinology, Fourth Clinical Medical College, Guangzhou University of Chinese Medicine, Shenzhen, China; ^3^Department of Cancer Immunology and Virology, Dana-Farber Cancer Institute, Boston, MA, United States; ^4^Department of Medicine, Harvard Medical School, Boston, MA, United States; ^5^Department of Clinical Laboratory, The Third Affiliated Hospital of Guangzhou Medical University, Guangzhou, China; ^6^Department of Encephalopathy First Affiliated Hospital of Henan University of Chinese Medicine, Zhengzhou, China; ^7^Institute of Clinical Pharmacology, Guangzhou University of Chinese Medicine, Guangzhou, China

**Keywords:** systems pharmacology, Kai-Xin-San, Alzheimer's disease, cholinergic system, neuroinflammation

## Abstract

Alzheimer's disease (AD) is a complex neurodegenerative disease characterized by cognitive dysfunction. Kai-Xin-San (KXS) is a traditional Chinese medicine (TCM) formula that has been used to treat AD patients for over a thousand years in China. However, the therapeutic mechanisms of KXS for treating AD have not been fully explored. Herein, we used a comprehensive network pharmacology approach to investigate the mechanism of action of KXS in the treatment of AD. This approach consists of construction of multiple networks and Gene Ontology enrichment and pathway analyses. Furthermore, animal experiments were performed to validate the predicted molecular mechanisms obtained from the systems pharmacology-based analysis. As a result, 50 chemicals in KXS and 39 AD-associated proteins were identified as major active compounds and targets, respectively. The therapeutic mechanisms of KXS in treating AD were primarily related to the regulation of four pathology modules, including amyloid beta metabolism, tau protein hyperphosphorylation process, cholinergic dysfunction, and inflammation. In scopolamine-induced cognitive dysfunction mice, we validated the anti-inflammatory effects of KXS on AD by determining the levels of inflammation cytokines including interleukin (IL)-6, IL-1β, and tumor necrosis factor (TNF)-α. We also found cholinergic system dysfunction amelioration of KXS is correlated with upregulation of the cholinergic receptor CHRNB2. In conclusion, our work proposes a comprehensive systems pharmacology approach to explore the underlying therapeutic mechanism of KXS for the treatment of AD.

## Introduction

Alzheimer's disease (AD), as the most common form of dementia, has become one of the leading causes of morbidity and mortality in the aged population. According to the World Alzheimer Report, up to 2019, there were over 50 million people living with dementia (Cao et al., 2018b; Gaugler and Al, 2019). Patients with AD suffer from a decline in learning and memory, cognitive deficits, and behavioral/personality changes, which lead to a heavy public health burden (Jia et al., 2018; Kumar and Tsao, 2019). As a complex multifactorial disease, AD is driven by extracellular deposition of beta amyloid (Aβ) and intracellular accumulation of tau protein. Current treatments can only provide limited symptomatic-relief benefits but fail to stop or reverse disease progression. Moreover, adverse effects, including diarrhea, nausea, and nightmares, further restrict the clinical treatment of AD (Masters et al., 2015). Therefore, there is an urgent need to discover novel therapeutic drugs with new mechanisms of action (MOAs) for treating AD.

Traditional Chinese medicine (TCM), which embraces centuries of knowledge and practical experience, has been used to treat many complex diseases in China for over 2,000 years (Cooper and Ma, 2017; Jiang et al., 2017). TCM has advantages for multi-targeting in intervention and treatment and has provided comprehensive prospects for understanding physiopathology and drug development for neurodegenerative diseases, including AD (Ho et al., 2010; Law et al., 2017). Kai-Xin-San (KXS) is a widely used TCM formula initially recorded in *Beiji Qianjin Yaofang* for treating dementia and depression in China since the Tang Dynasty. It is comprised of four herbs: *Panax ginseng C. A. Mey* (RENSHEN, RS), *Polygala tenuifolia Willd* (YUANZHI, YZ), *Acorus tatarinowii* (SHICHANGPU, SCP), and *Poria* (FULING, FL) (Cao et al., 2018a). Previous studies of KXS mainly focused on the mechanism of a single target-oriented pathway or neurotransmitter regulation, which cannot comprehensively illuminate the therapeutic effects and mechanism of action (MOA) of KXS for AD treatment (Lu et al., 2017; Wang et al., 2017; Cao et al., 2018a; Gao et al., 2018). Herein, there is a need to investigate the overall beneficial effects of KXS for treating AD using advanced approaches.

**Figure 1:**
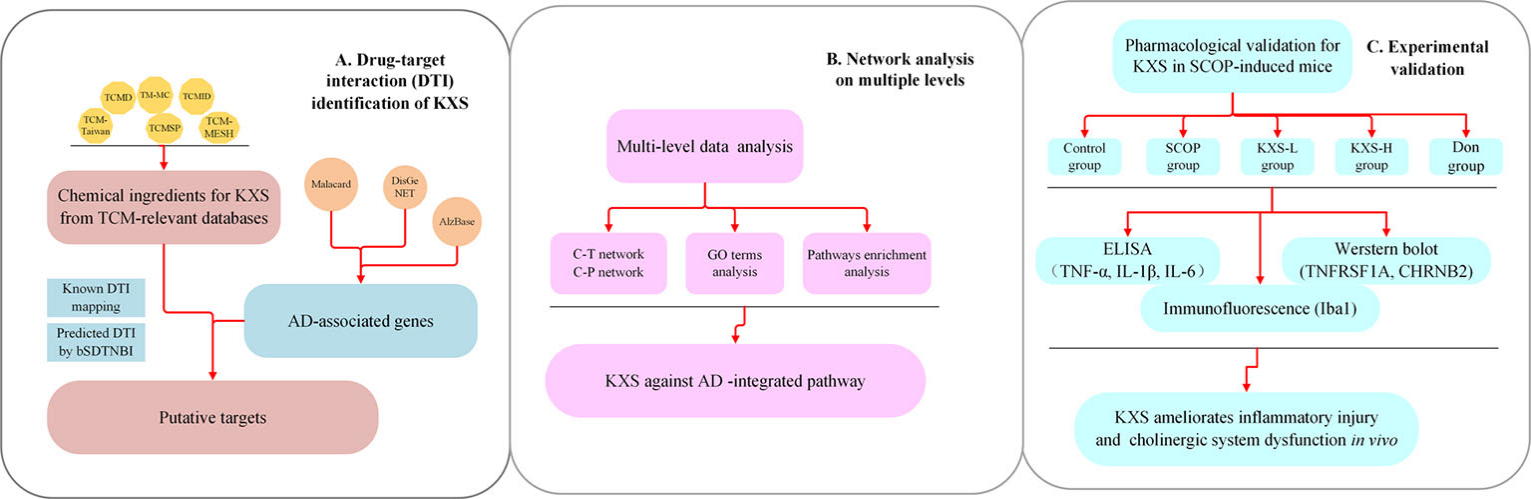
Flowchart of the systems pharmacology approach for deciphering the therapeutic mechanisms of action of Kai-Xin-San (KXS) on Alzheimer's disease (AD). **(A)** Drug-target interaction (DTI) identification. **(B)** Network analysis of multiple data to investigate the therapeutic mechanisms of KXS on AD. **(C)** Experimental validation *in vivo* to explore the pharmacological mechanisms of KXS on AD.

Systems pharmacology is a cutting-edge methodology that combines computational and experimental tools toward discovering novel therapeutic agents and understanding the therapeutic mechanisms of complex diseases. (Fang et al., 2017a; Fang et al., 2019). In recent years, systems pharmacology-based approaches have provided new insights into elucidating the mechanisms of TCM in the treatment of diseases such as cardiovascular diseases and AD (Zhou and Wang, 2014; Fang et al., 2017a; Cai et al., 2018). In this study, we employed a systems pharmacology approach to identify potential compounds, candidate targets, and therapeutic mechanisms of KXS against AD disease from a holistic prospect (**Figure 1**). Briefly, we first determined the comprehensive AD-associated genes and ingredients of KXS after integrating different data sources. We further predicted candidate targets based on a balanced substructure-drug-target network-based inference approach (bSDTNBI). Subsequently, the targets of KXS were mapped onto AD-relevant genes to determine their biological functions and corresponding AD pathways. Furthermore, we performed multiple level data analyses to reveal the MOA of KXS on AD treatment. Finally, we validated the proposed pharmacological mechanism of KXS in a scopolamine (SCOP)-induced AD mouse model.

## Materials and Methods

### AD-Associated Gene Collection

Genes related to AD were collected from several public disease gene-related databases, including Malacard (https://www.malacards.org), DisGeNet database, GWAS catalog, HGMD (Pinero et al., 2017), AlzBase database (http://alz.big.ac.cn/alzBase/summary/Gene), and AlzPlatform. AlzPlatform is an AD-specific chemogenomics knowledgebase for target identification and drug discovery (Liu et al., 2014). Ultimately, a total of 447 AD-associated genes were obtained (**Supplementary Datasheet S1**).

### KXS Ingredient Collection

All ingredients in KXS (4 herbs) were collected from six TCM-related databases, including TCMID (Xue et al., 2013), TCM-Taiwan (Chen, 2011), TCMD (He et al., 2001), TCMSP (Ru et al., 2014), TM-MC (Kim et al., 2015), and TCM-MESH (Zhang et al., 2017). For each database, we extracted the chemical structures of each herb as an SDF file. Subsequently, six SDF files were merged to a single SDF, which contained all of the chemical structures from the six data sources. SMILES as well as InChIKey were generated by Open Babel (O'boyle et al., 2011) for each ingredient. After removing the duplicates, 1,118 ingredients in KXS were finally obtained.

### Target Identification for KXS

In this work, the known targets of KXS were extracted from our previous integrated database (Fang et al., 2017b), which contains 7,314 drug-target interactions (DTIs) connecting 751 targets and 2,388 natural products.

In a previous study, we developed predictive network models to identify new targets of natural products *via* the bSDTNBI approach (Fang et al., 2017b), which prioritized potential targets for known drugs and new chemical entities (NCEs) by resource-diffusion processes of the substructure drug-target network. During the course, two parameters including α and β were imported to balance the initial resource allocation of different node types as well as the weighted values of different edge types. Moreover, parameter γ was also utilized to balance the influence of hub nodes. The final parameters (α = β =0.1, γ = –0.5, and k = 2) of bSDTNBI were adopted from a previous study (Wu et al., 2016). Among the four network models developed using different types of fingerprints, bSDTNBI_KR performed best with the highest value of P (0.049), R (0.752), eP (27.02), eR (27.24), and AUC (0.959). Thus, bSDTNBI_KR was utilized to predict new targets of natural products in the global network.

### Network Construction

To comprehensively understand the complex interactions among herbs, compounds, targets, and pathways, herb-target and compound-target networks were constructed by Cytoscape (version 3.2.1) and Gephi (version 0.9.2). In the graphical network, the nodes represent compounds, targets, or herbs, while the edges denote links among them. The quantitative property “degree” was calculated as the number of edges linked to each node, indicating the importance of a given node in a network.

### Gene Ontology (GO) Enrichment Analysis

The biological significance of protein targets can be interpreted *via* GO enrichment analysis. In this work, we conducted a biological process (BP) interpretation by mapping AD-related genes (with a degree greater than 2) of KXS to the DAVID database (https://david.ncifcrf.gov/home.jsp) (Huang Da et al., 2009). DAVID is an integrated biological knowledgebase and analytic tool aimed at systematically extracting the biological meaning from large gene/protein lists.

### Pathway Construction and Analysis

To investigate the roles of protein targets in the pathophysiological network of AD and how KXS acts on AD by regulating certain pathways, an “AD-integrated pathway” was proposed based on our present understanding of AD pathology and target identification. In brief, the protein targets were first mapped to the Kyoto Encyclopedia of Genes and Genomes database (KEGG, http://www.genome.jp/kegg/) to obtain the potential pathways. Subsequently, pathways related to AD pathological processes were selected and incorporated into an “AD-integrated pathway” to analyze the therapeutic mechanisms of KXS for treating AD.

### Experimental Validation

#### KXS Preparation

The four herbs in KXS (RS, FL, SCP, and YZ) were obtained from the First Affiliated Hospital of Guangzhou University of Chinese Medicine (Guangzhou, China) and were mixed at a ratio of 3:3:2:2 referenced the previous studies (Cao et al., 2012; Xu et al., 2019). The extraction process and quality control were implemented according to our previous study (Xu et al., 2019). Briefly, SCP was added to six parts of water as a solvent for 2 h, followed by heat reflux extraction for 8 h. Volatile oil and SCP dregs were saved. RS was extracted twice by 60% ethanol as solvent for each time with 1 hour. The extracts were combined and filtrated, and the RS dregs were saved. The dregs of SCP, RS, FL, and YZ were added to 10 parts of water and extracted twice, for 1 h each. All extracts were combined and evaporated on a rotary evaporator. Finally, volatile oil of SCP and original liquid after concentrated were mixed and refrigerated at −20°C for usage.

#### AD Model and Drug Treatments

Kun-Ming mice (8 weeks old) weighing 30–35 g were obtained from Sibeifu Biotechnology (Beijing, China) and housed at 22 ± 2°C, with a relative humidity of 55% ± 5%, a 12 h light/dark cycle and *ad libitum* access to food and water. All animal procedures were performed in accordance with the principles and guidelines of the National Institutes of Health Guide for the Care and Use of Laboratory Animals and approved by the Guangzhou University of Chinese Medicine Animal Ethics Committee.

AD model was induced by intraperitoneally injected SCOP (3 mg/kg) for a consecutively week. In this week, Morris water maze and new objection recognition test were performed daily after 30 min of SCOP injection to evaluate animal model. In this study: mice were randomly allotted to five groups: Control group (n=10; 0.9% saline p.o.+ 0.9% saline, i.p.), SCOP group (n=10; 0.9% saline p.o.+ SCOP 3 mg/kg/d i.p.), low-dose KXS group (n=10; KXS 1.4 g/kg/d p.o.+ SCOP 3 mg/kg/d i.p.), high-dose KXS group (n=10; KXS 2.8 g/kg/d p.o.+ SCOP 3 mg/kg/d i.p.), and Don group (n=10; donepezil 3 mg/kg/d p.o.+ SCOP 3 mg/kg/d i.p.). The mice were orally administered 0.9% saline, KXS, or donepezil mice for 14 continuous days. Memory impairment was induced by SCOP treatment (3 mg/kg body weight) for 7 days.

#### Western Blot Analysis

The hippocampus and cortex were homogenized in SDS lysis buffer containing a protease inhibitor and phosphatase inhibitor mixture (Sigma-Aldrich). After sonication, lysates were centrifuged at 3,000×g and 4°C for 15 min. Supernatants were collected, and protein concentrations were determined by the Bradford assay (Bio-Rad). The same amounts of proteins were resolved on SDS-PAGE gels, transferred to PVDF membranes (Millipore), and probed with primary antibodies overnight at 4°C. Rabbit anti-TNFRSF1A (1:1,000, Abcam), anti-CHRNB2 (1:1,000, Abcam), and anti-β-actin (1:10,000, Abcam) were used as the primary antibodies. Immunoblots were visualized by the ECL western blot detection kit (Millipore) and quantified by densitometry and ImageJ software (National Institutes of Health) (Lee et al., 2017).

#### Immunofluorescence

Mice were transcardially perfused with 0.9% saline. The mouse brains were fixed in 4% paraformaldehyde (PFA) for 72 h, followed by dehydration with different concentrations of ethanol and paraffin embedding. A paraffin microtome was used to cut 5-μm-thick coronal brain sections.

For immunofluorescence, sections were deparaffinized by ethanol and incubated with 3% H_2_O_2_. Antigen retrieval was performed by heating the sections in sodium citrate buffer (10 mM trisodium citrate, 0.5% Tween-20 in H_2_O, pH 6.0) at 70°C for 30 min. Sections were then permeabilized and incubated with blocking solution containing the Iba-1 antibody (1:100, Abcam) overnight at 4°C. The sections were then stained with a fluorophore-conjugated secondary antibody (1:1,000, CST) for 1 h, followed by 4′6-diamino- 2-phenylindole (DAPI, Sigma) staining for another 1 h. Images were acquired by a fluorescence microscope Model DMi8 (Leica, Germany) and quantified using ImageJ software (National Institutes of Health) (Muhammad et al., 2019).

#### ELISA Analysis

Mice cortical and hippocampal tissues were sequentially homogenized in ice-cold PBS, and the supernatants were centrifuged at 21,000×g for 20 min at 4°C. The levels of IL-1β, IL-6, and TNF-α were measured using an enzyme-linked immunosorbent assay (ELISA, Biological Technology, Jiangsu) (Karthivashan et al., 2018) according to the manufacturer's instructions.

#### Statistical Analysis

All data were expressed as means ± SEM of at least three independent experiments. Statistical analyses were performed using SPSS (version 20.0, IBM, Armonk, NY). Statistical tests between multiple datasets were carried using a one-way analysis of variance (ANOVA) followed by Dunnett's *post hoc* test to determine statistical significance, as appropriate. A *P* value < 0.05 was considered statistically significant.

## Results and Discussion

### Collection of Chemical Ingredients in KXS

In the present study, a total of 1,118 compounds in KXS were collected from specific TCM-relevant databases after removing the duplicate structures. The numbers of ingredients for each herb in KXS were 628 (RS), 237 (YZ), 119 (FL), and 210 (SCP). Among the 1,118 chemical ingredients, there were 70 compounds that existed in more than one herb. For example, compound M449 (palmitic acid) could be found in all four herbs in the KXS formula. Candidate ingredients were defined if there were known targets or putative targets *via* bSTDNBI for a certain ingredient. Ultimately, 1,113 candidate compounds were obtained (**Supplementary Datasheet S1**).

### Identification of Putative Targets for the Ingredients in KXS

After merging the known DTIs and predicted DTIs by bSTDNBI, we identified 439 target proteins for 1,113 candidate compounds. We further identified 39 AD-associated targets for KXS by overlapping 439 potential targets into the curated human AD-associated 447 genes. Detailed information of the 39 AD-associated targets can be found in **Supplementary Datasheet S1**.

### Analysis of the Synergetic Actions of KXS Against AD

The TCM theory “Jun-Chen-Zuo-Shi” serves as the guide for physicians when formulating herbal prescriptions. Among the herbs in KXS, RS and FL serve as the “Jun” and “Chen” herbs to treat the major symptoms and signs of AD, while YZ and SCP act as the “Zuo” and “Shi” herbs for improving the therapeutic effects of the “Jun” and “Chen” herbs and guiding the herbs to the disease targets (Cao et al., 2018a). Herein, we investigated the distribution of AD-relevant targets among the four herbs (18 from RS, 9 from FL, 16 from SCP, 15 from YZ) *via* a Venn analysis (**Supplementary Datasheet S1**).

**Figure 2:**
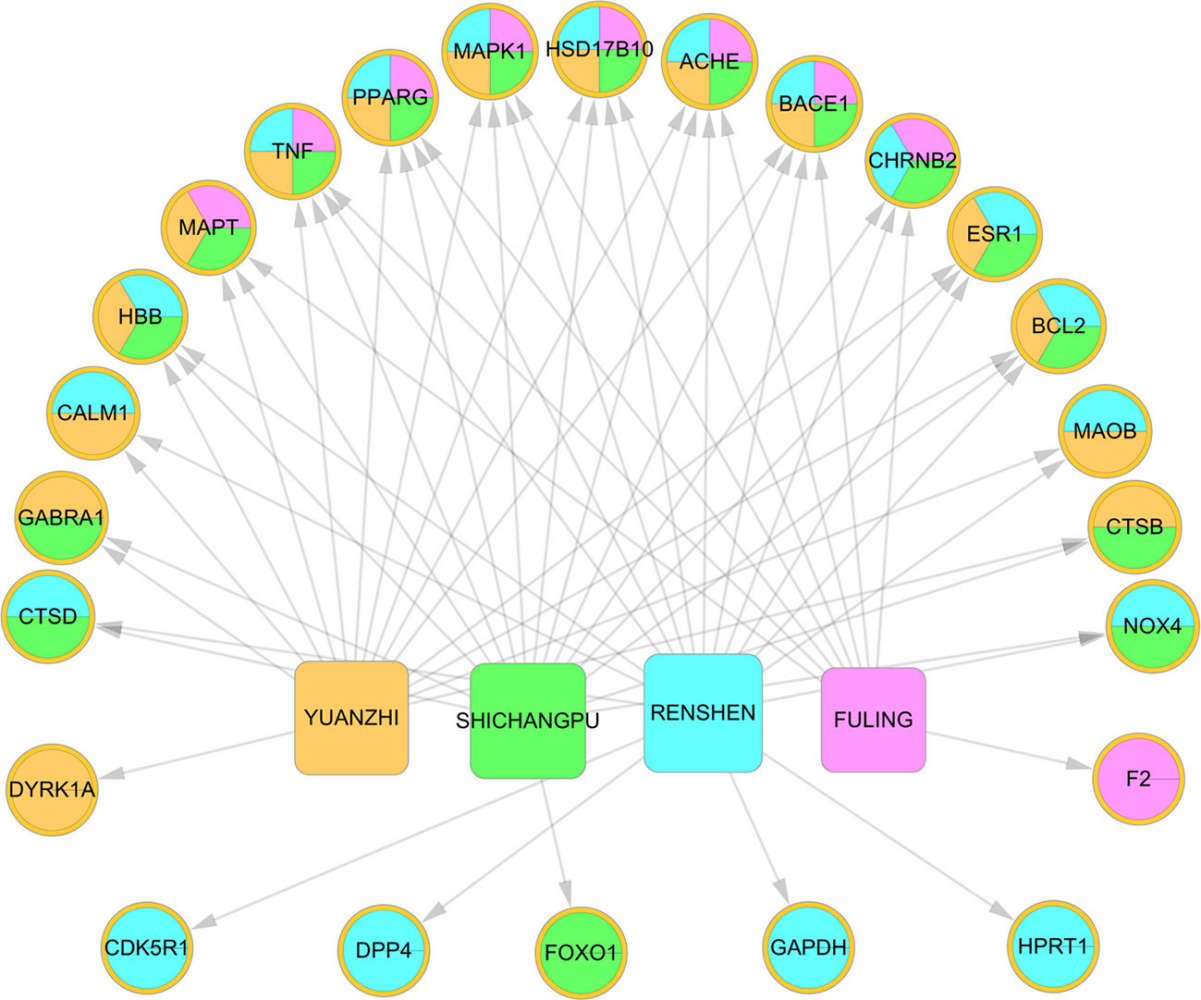
Herb-target network of Kai-Xin-San (KXS). The ellipses represent the targets of KXS. The round rectangle indicates the herbs in KXS.

The four herbs covered all 39 AD-associated targets (**Figure S1**). Among the four herbs, RS covered the largest number (18) of AD targets for the “Jun” herb in KXS for treating AD, demonstrating the consistency of TCM theory. To exploit the synergistic MOAs of the KXS formula at the individual herb level, we constructed a herb-target network (**Figure 2**, H-T network). We found that the four herbs in KXS shared six common targets, suggesting that KXS could exert its magnifying effects by targeting these key targets. These targets include acetylcholinesterase (ACHE), beta-secretase 1 (BACE1), hydroxysteroid 17-beta dehydrogenase 10 (HSD17B10), mitogen-activated protein kinase 1 (MAPK1), peroxisome proliferator-activated receptor gamma (PPARG), and tumor necrosis factor (TNF). BACE plays a key role in neurotoxic Aβ generation (Moussa-Pacha et al., 2019). Tumor necrosis factor-α (TNF-α) has been confirmed to advance Aβ production through enhancing the expression of BACE1 and suppressing the clearance of Aβ (Yamamoto et al., 2007; Decourt et al., 2017). Moreover, ACHE regulates acetylcholine in the cholinergic system, which plays a role in the learning process in AD patients (Ferreira-Vieira et al., 2016). Therefore, it is likely that the four herbs in KXS are able to regulate several important AD-associated pathological processes to exert advanced or synergistic effects in AD intervention and treatment.

### Compound-Target (C-T) Network Analysis

**Figure 3:**
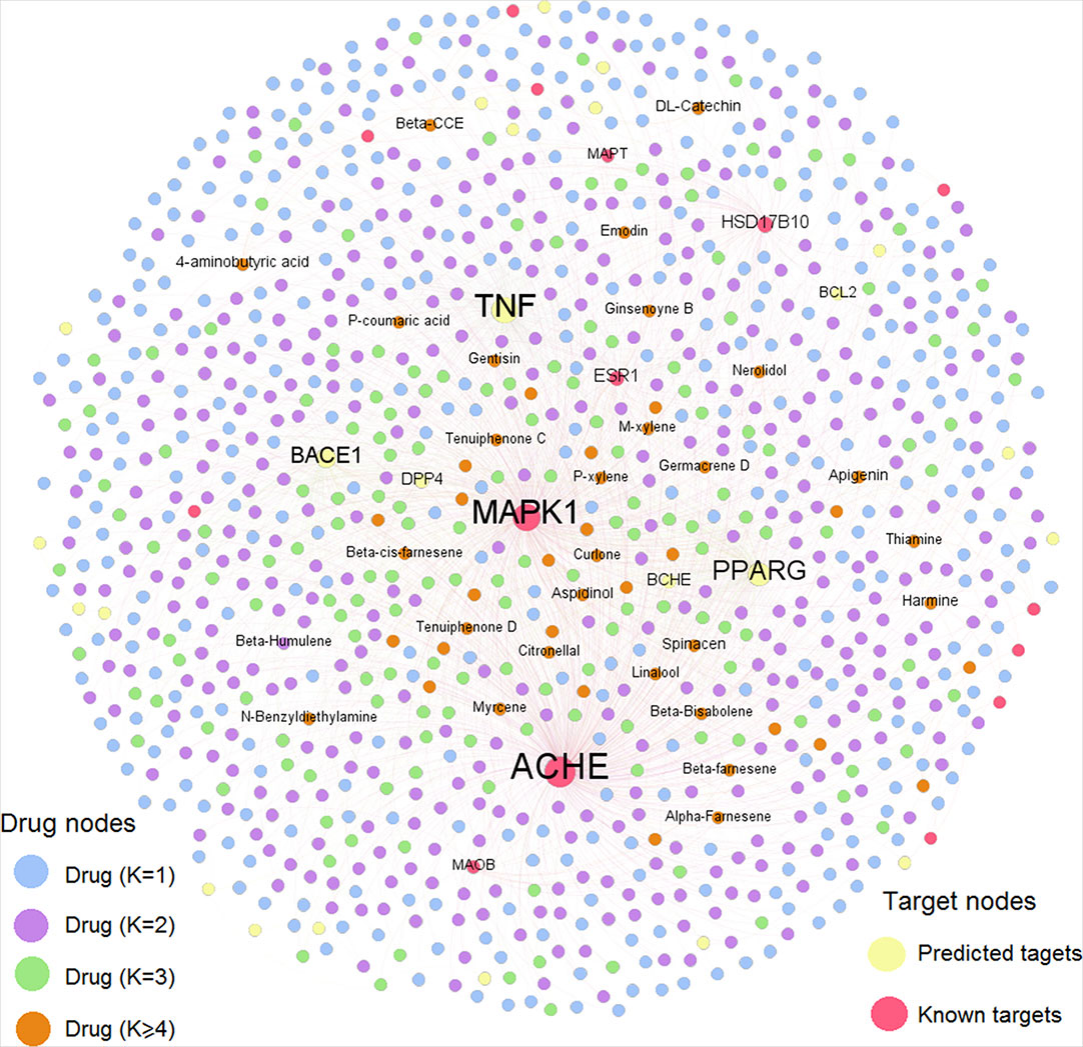
Global compound-target network of the candidate ingredients of Kai-Xin-San (KXS).

To further decipher the therapeutic mechanism of KXS against AD, we constructed a AD-specific compound-target (C-T) network consisting of 1,936 C-T interactions (**Figure 3**). The C-T network comprises 40 known CTIs and 1,896 predicted CTIs connecting 1,021 compounds to 39 AD target proteins. Among the 1,021 ingredients, nine have a target degree (*N*) higher than five, including M909 (apigenin, *N*=9), M638 (4-aminobutyric acid, *N*=6), M326 (aspidinol, *N*=5), M429 (dodecanal, *N*=5), M621 (harmine, *N*=5), M8 (spinacen, *N*=5), and M903 (DL-catechin, *N*=5). For the 39 AD targets, five were targeted by more than 100 compounds (*D*): ACHE (*D*=454), MAPK1 (*D*=372), TNF (*D*=346), PPARG (*D*=261), and BACE1 (*D*=174). Previous studies have demonstrated that these targets are beneficial for AD patients. For example, inhibitors of ACHE (e.g., donepezil) can provide modest symptomatic relief for AD patients (Arvanitakis et al., 2019). Recently, both preclinical and clinical studies have indicated that PPARG agonists can improve learning and memory abilities in AD patients (Khan et al., 2019). BACE1 inhibitors have also been reported to repress the generation of neurotoxic amyloid protein (Dobrowolska Zakaria and Vassar, 2018; Moussa-Pacha et al., 2019). Overall, this AD-specific C-T network contributes to uncovering the MOA of chemicals in KXS against AD. The detailed information of the C-T network is provided in **Supplementary Datasheet S2**.

### GO BP Enrichment Analysis

**Figure 4:**
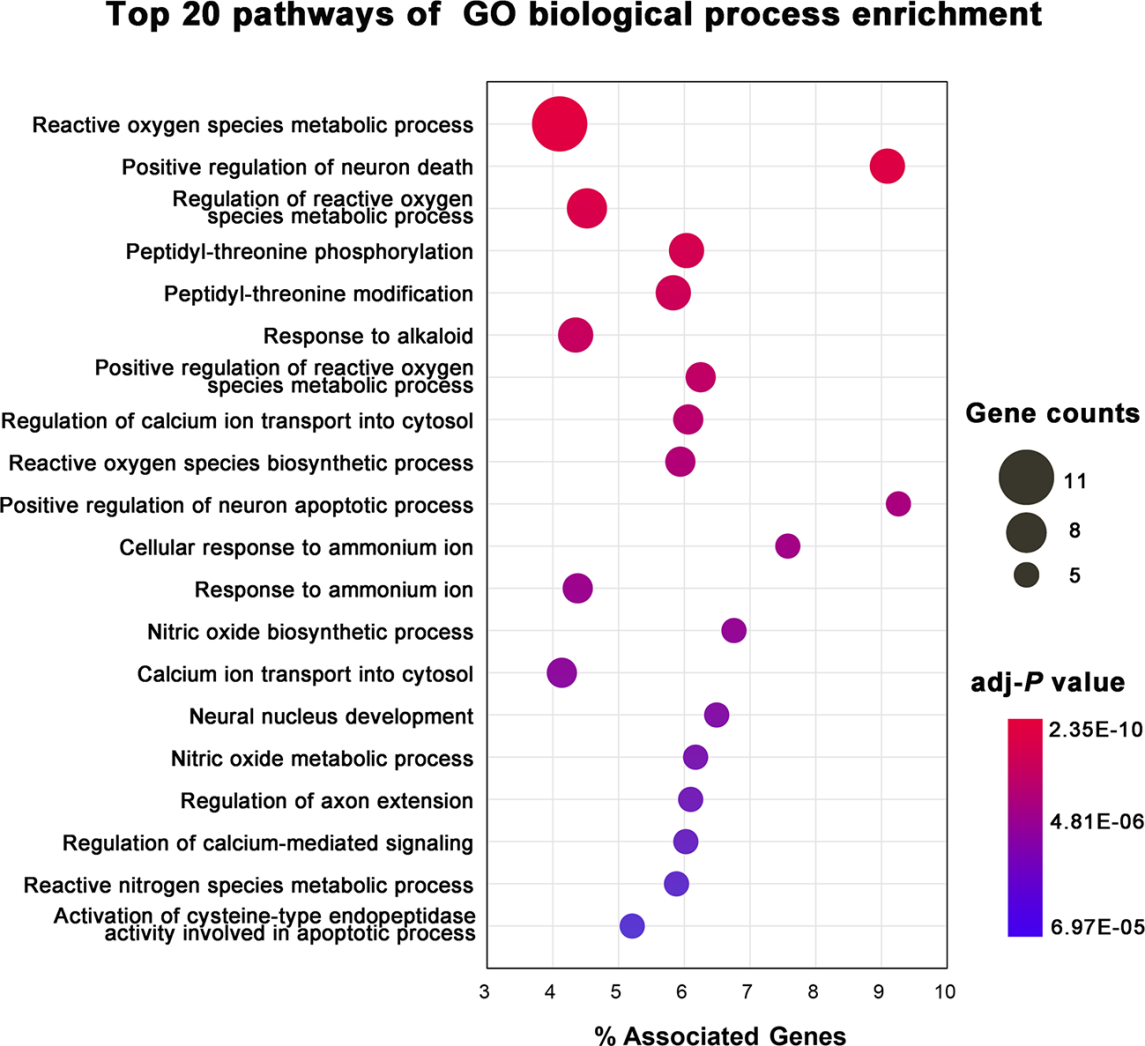
Gene Ontology (GO) biological process enrichment analysis of targets of Kai-Xin-San (KXS) for the treatment of Alzheimer's disease (AD).

To illustrate the related signaling pathways involved in the treatment of AD with KXS, GO BP enrichment analysis was performed using ClueGO, a plug in of the Cytoscape software package. Only the top 20 significantly enriched (adjusted *P* < 0.05) signaling pathways were preserved for further analysis. Multiple signaling pathways were involved in the treatment of AD with KXS, including reactive oxygen species metabolic process, positive regulation of neuron death (apoptotic process), regulation of calcium ion transport into the cytosol, and nitric oxide biosynthetic process, etc. (**Figure 4**). These signaling pathways seem to play vital roles in AD-associated pathological processes. For instance, Aβ-induced neuron death is considered a central pathway in the pathogenesis of AD through oxidative stress, inflammation, apoptosis, or autophagy (Cao et al., 2018b; Kumar and Tsao, 2019).

### Integrated Pathway Analysis

**Figure 5:**
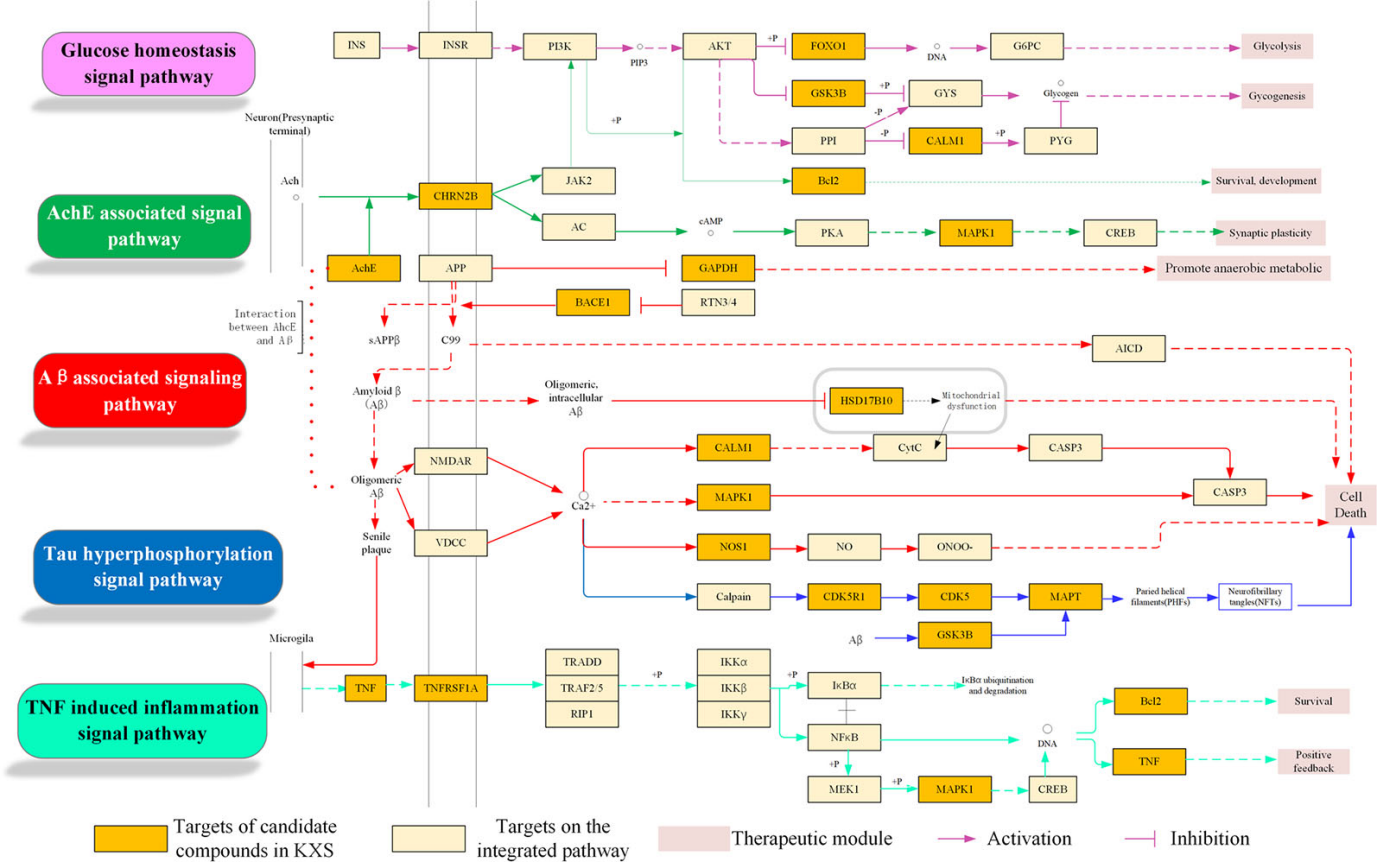
Integrated Alzheimer's disease (AD)-pathway and Kai-Xin-San (KXS) action in the therapeutic modules.

In this analysis, pathways directly related to AD were incorporated into an “AD-integrated pathway” based on target identification for KXS and AD pathology, including the glucose homeostasis signaling pathway, ACHE-associated signaling pathway, Aβ-associated signaling pathway, tau hyperphosphorylated signaling pathway, and TNF-induced inflammation signaling pathway. As depicted in **Figure 5**, several pathophysiological modules were involved in this integrated pathway, such as glycolysis, cell death, survival, and synaptic plasticity. Here, four representative modules will be discussed to illustrate the underlying therapeutic mechanisms of KXS for AD treatment.

#### Aβ-Associated Pathological Signaling Pathway Regulation Module

It is well known that abnormal Aβ aggregation and accumulation plays a critical role in the pathogenesis of AD. Amyloid precursor protein (APP) is cleaved by α-secretase, β-secretase, and γ-secretase *via* non-amyloidogenic and amyloidogenic pathways. In the non-amyloidogenic pathway, proteolysis of APP by α- and γ-secretases yields nonpathogenic fragments, including sAPPα and C-terminal fragments. However, the amyloidogenic pathway involves cleavage by β- and γ-secretases, leading to the production of sAPPβ, C-terminal fragments, and Aβs (Barage and Sonawane, 2015; Selkoe and Hardy, 2016). Accumulated Aβ can further directly or indirectly trigger other pathological processes, such as mitochondrial dysfunction and inflammation, inducing neuronal death in AD (Mao et al., 2012). Thus, targeting β- and γ-secretases is regarded as a primary therapeutic approach for reducing Aβ production in AD patients. As shown in **Figure 5**, KXS targets key proteins implicated in the Aβ-associated signaling pathway, including BACE1 (β-secretase 1), GAPDH (glycolytic glyceraldehyde-3-phosphate dehydrogenase), and SD17B10 (hydroxysteroid 17-beta dehydrogenase 10). GAPDH (glycolytic glyceraldehyde-3-phosphate dehydrogenase), an abundantly expressed oxidoreductase for glucose metabolism, has been reported to possess diverse non-glycolytic functions, including interaction with Aβ (El Kadmiri et al., 2014; Sen et al., 2018). Target prediction suggested that M693 (citric acid) could bind with GAPDH, which might alleviate Aβ accumulation by disturbing the cross-talk between Aβ and GAPDH (Gerszon and Rodacka, 2018). In addition, HSD17B10 (hydroxysteroid 17-beta dehydrogenase 10), a hydroxysteroid dehydrogenase localized in the mitochondria (He et al., 2018), was the potential target of the compounds in KXS, including M150, M388, and M500. This target has been recognized as a putative intracellular mediator of Aβ neurotoxicity *via* its dozen proteins that bind to Aβ (Reddy et al., 2012). Thus, KXS could disturb the combination of Aβ and HSD17B10 to alleviate mitochondria dysfunction as it activates β-secretase and γ-secretase and facilitates Aβ generation (Yang et al., 2014). Taken together, KXS might prevent and treat AD by regulating Aβ generation and aggregation.

#### Neurofibrillary Tangles Regulation Module

NFTs (neurofibrillary tangles), aggregating by abnormal hyperphosphorylation of microtubule-associated protein tau (MAPT) in the brain, correlate well with the severity of cognitive deficits in AD patients (Simic et al., 2016; Jouanne et al., 2017). Tau protein can be hyperphosphorylated by various kinases including CDK5 (cyclin-dependent kinase 5) (Zhao et al., 2019) and GSK3B (glycogen synthase kinase 3beta) (Chu et al., 2017). Therefore, a reduction in tau protein levels (Wang and Mandelkow, 2015) as well as inhibition of tau protein hyperphosphorylation and aggregation are potential therapeutic avenues for AD treatment. As shown in **Figure 5**, KXS was predicted to target three crucial proteins (i.e., CDK5, GSK3B, and MAPT) involved in the regulation of NFTs. For example, the compounds M621, M622, and M909 were predicted to interact with CDK5 and GSK3B. Emerging evidence has revealed that inhibiting these kinases can prevent tau protein hyperphosphorylation and NFT formation (Congdon and Sigurdsson, 2018). Additionally, compounds such as M84, M257, and M686 were identified to bind to MAPT, suggesting that KXS might decrease tau protein levels to alleviate NFT formation and ameliorate Aβ-dependent neurotoxicity (Peters et al., 2019).

#### Cholinergic System Dysfunction Regulation Module

The loss of cholinergic neurons in AD patients leads to learning and memory deficits (Ferreira-Vieira et al., 2016). Cholinesterase inhibitors can prevent the breakdown of acetylcholine and preserve its activity at cholinergic synapses (Hampel et al., 2018). **Figure 5** shows that the KXS formula regulates the cholinergic system signaling pathway by targeting ACHE (acetylcholinesterase) and CHRN2B (cholinergic receptor nicotinic beta 2 subunit). Four compounds, M21, M389, M597, and M944, interact with ACHE and CHRN2B, which might improve the level of acetylcholine and sustain its activity. Our previous study demonstrated that KXS could ameliorate cognitive function in SCOP-induced mice by regulating the cholinergic system through sustaining ACh levels, increasing ChAT activity, and decreasing AChE activity (Xu et al., 2019), which is in accordance with our systems pharmacology-based analysis.

#### Neuroinflammation Regulation Module

Neuroinflammation refers to the inflammatory response in the central nervous system secondary to neuronal insult (Calsolaro and Edison, 2016). Activating microglia and astrocytes in AD leads to the production and release of inflammatory cytokines, including interleukin-6 (IL-6), interleukin-1β (IL-1β), and TNF-α (Heneka et al., 2015; Kinney et al., 2018; Ozben and Ozben, 2019). **Figure 5** suggests that KXS, a more specifically the compounds M230, M885, and M606, could modulate neuroinflammation in AD by regulating TNF-α and its receptor TNF-receptor superfamily 1A (TNFRSF1A). TNF-α can exacerbate Aβ burden by increasing γ-secretase activity and β-secretase production (Cheng et al., 2014; Chang et al., 2017) or bind to TNFRSF1A. This effect is partly mediated through MAPK1 signaling, which leads to the expression of molecules that participate in inflammation and amyloid genesis (Montgomery and Bowers, 2012; Decourt et al., 2017). Furthermore, IL-1β and IL-6 are able to enhance the amyloidogenic process of APP (Tachida et al., 2008) and the hyperphosphorylation of tau epitopes (Kitazawa et al., 2011). Suppression of either IL-1β or IL-6 can ameliorate neuroinflammation and neurodegeneration (Basu et al., 2004; Rothaug et al., 2016). In summary, KXS might prevent and treat AD by regulating neuroinflammation.

### Experimental Validation of KXS in SCOP-Induced Mice

#### KXS Ameliorates Inflammatory Injury in SCOP-Induced Mice

The systems pharmacology analysis indicated that the therapeutic mechanism of KXS for AD involves several potential mechanisms, including the regulation of cholinergic system dysfunction and neuroinflammation.

**Figure 6:**
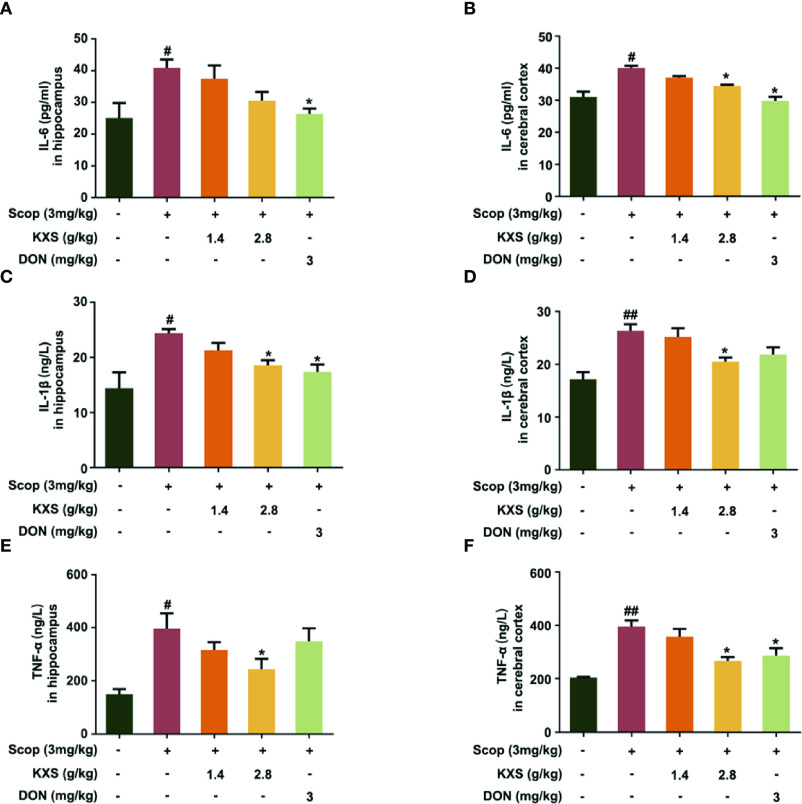
Kai-Xin-San (KXS) attenuates inflammatory injury in scopolamine (SCOP)-induced mice. The levels of interleukin (IL)-6, IL-1β, and tumor necrosis factor (TNF)-α in the hippocampus **(A, C, E)** and cortex **(B, D, F)** were determined by ELISA. Data are expressed as means ± SEM. Difference was calculated using one-way ANOVA followed by Dunnett's *post hoc* test (n=5, ^#^*P* < 0.05, ^##^*P* < 0.01 the control group versus the SCOP group; *P < 0.05 versus the SCOP group).

Chronic neuroinflammation (sustained microglial activation and overproduction of pro-inflammatory cytokines) has been implicated in the pathophysiology of AD. To validate the mechanism of KXS on neuroinflammation *in vivo*, we measured proinflammatory cytokine levels in SCOP-induced mice treated with KXS. As shown in **Figure 6**, SCOP administration significantly increased the levels of TNF-α, IL-1β, and IL-6 compared to the non-treated control group (*P* < 0.05 or *P* < 0.01). However, KXS and Don significantly (*P* < 0.05) attenuated the upregulation of IL-1β, TNF-α, and IL-6 compared with the SCOP alone group both in the hippocampus and cortex.

**Figure 7:**
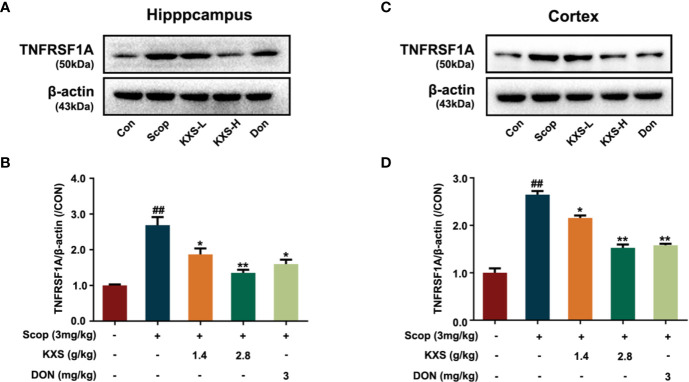
Kai-Xin-San (KXS) decreases the protein expression of tumor necrosis factor receptor superfamily member 1A (TNFRSF1A) in scopolamine (SCOP)-induced mice. Western blot analysis showing the protein expression levels of TNFRSF1A in the hippocampus **(A)** and cortex **(C)**. Quantiﬁcation of TNFRSF1A levels in panel **(A, B)** or **(C, D)** was normalized to that of actin. Data represent the means ± SEM (n=3, ^##^*P* < 0.01 versus the control group; *P < 0.05, **P < 0.01 versus the SCOP group).

TNFRSF1A is the main cell surface receptor for TNF. Next, we explored the expression of TNFRSF1A by western blotting. As shown in **Figure 7**, the protein expression of TNFRSF1A increased in the SCOP group in both the hippocampus and cortex (*P* < 0.01, vs. the control group). Both the low-dose and high-dose KXS groups and the DON wed group decreased TNFRSF1A expression (*P* < 0.05 or *P* < 0.01, vs. the SCOP group).

**Figure 8:**
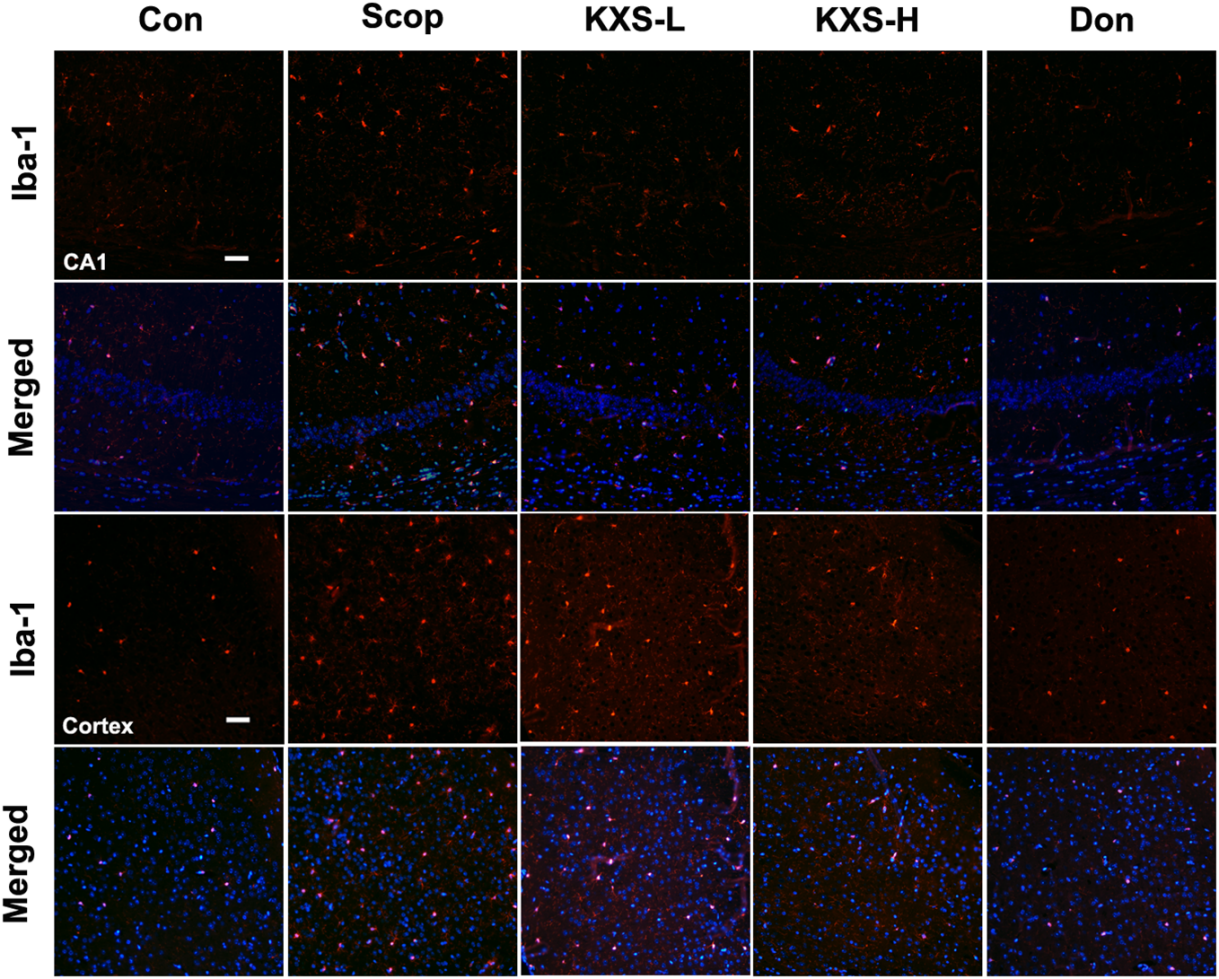
Kai-Xin-San (KXS) attenuates microglia activation in scopolamine (SCOP)-induced mice. Immunofluorescence analysis in the hippocampus (CA1) and cortex. Microglia were stained with anti-Iba-1 (red) and the nuclei were stained with DAPI (blue). Scale bar: 50 mm. Con, control group; SCOP, scopolamine; KXS-L, low-dose Kai-Xin San (1.4 g/kg); KSX-H, high-dose Kai-Xin San (2.8 g/kg); Don, donepezil.

Microglia secrete a number of inflammatory cytokines including TNF-α, IL-1β, and IL-6 in response to inflammatory stimuli (Ozben and Ozben, 2019). To further probe the effect of KXS on neuroinflammation, we detected the expression of Iba-1, a microglia activation marker, in the hippocampus and cortex using immunofluorescence. As displayed in **Figure 8**, elevated microglia activation was observed in the SCOP group in the hippocampus (CA1) and cortex, whereas KXS and donepezil markedly attenuated microglia activation. These results demonstrate that KXS inhibits neuroinflammation in SCOP-induced mice

#### KXS Attenuates Cholinergic System Dysfunction in SCOP-Induced Mice

**Figure 9:**
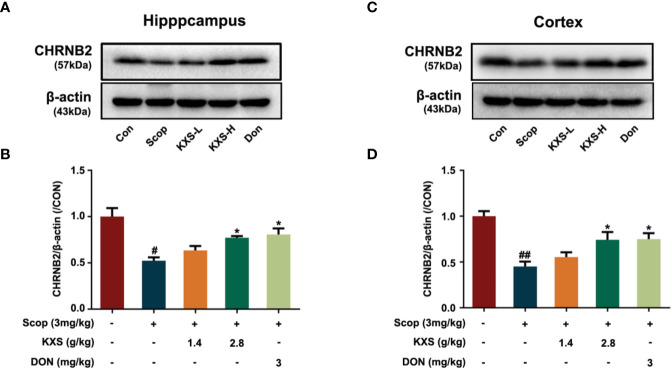
Kai-Xin-San (KXS) increases the expression of nicotinic acetylcholine receptor beta 2 (CHRNB2) in scopolamine (SCOP)-induce mice. Western blot analysis of the protein expression of CHRNB2 in the hippocampus **(A)** and cortex **(C)**. Quantiﬁcation of CHRNB2 level in panel **(A, B)** or **(C, D)** was normalized to that of actin. Data represent the means ± SEM (n=3, ^#^*P* < 0.05, ^##^*P* < 0.01 versus the control group; *P < 0.05 versus the SCOP group).

In our previous study, we demonstrated that KXS could alleviate cholinergic system dysfunction in SCOP-induced mice by increasing ChAT activity and the Ach content, and decreasing AChE activity (Xu et al., 2019). However, the detailed mechanism remains unclear. In this study, a systems pharmacology-based analysis suggested that KXS could regulate the expression of the target protein CHRNB2 (a nicotinic acetylcholine receptor). CHRNB2 is lost in the brains of AD patients and knockout of the CHRNB2 gene impairs neuronal survival in aging (Cook et al., 2004). Therefore, we further investigated the effect of KXS on cholinergic dysfunction *via* CHRNB2 expression in SCOP-induced mice. As shown in **Figure 9**, SCOP significantly downregulated the expression of CHRNB2 compared to the control group in both the hippocampus and cortex (*P* < 0.05, *P* < 0.01, respectively). High-dose KXS and Donepezil remarkably increased the expression of CHRNB2 both in the hippocampus and cortex (*P* < 0.05, vs. the SCOP group). These data suggest that CHRNB2 may play an important role in the effects of KXS ameliorating cholinergic system dysfunction of SCOP-induced mice.

## Conclusion

To date, acetylcholinesterase inhibitors and NMDA receptor antagonists have been the primary choice for treating AD patients, showing marginal benefits for alleviating symptoms. The side effects are not ignorable and add to the ongoing AD burden. Therefore, it is urgent that novel and efficient curative remedies are discovered for AD patients. TCM efficacy in medical practice has been demonstrated for thousands of years. In contrast to western medicine, TCM formulas emphasize the regulation of interactions among all illness-associated elements within the abnormal body toward a balanced/normal condition. However, as TCM formulas contain hundreds of chemical components, the specific pharmacological mechanisms through which TCM prescriptions exert their effects against diseases are still difficult to illustrate.

KXS, a TCM formula that has been used for thousands of years in China to treat cognitive dysfunction, has been shown to improve learning and memory in animals studies. In this study, we developed an integrative systems pharmacology approach to illustrate the therapeutic mechanisms of KXS against AD. For the first time, we identified 39 AD-associated targets of KXS ingredients using known target mapping and *in silico* target prediction. Furthermore, we deciphered potential MOAs of KXS in AD treatment *via* a multiple data integration analysis, including an herb-target network analysis, compound-target network analysis, synergic action analysis, and integrated pathway analysis. Our systems pharmacology analysis was validated in *in vivo* experiments. The results showed that KXS could ameliorate cognitive dysfunction mainly through inhibiting the inflammation of microglia in SCOP-induced mice. More importantly, we found that the therapeutic effect of alleviating cholinergic system dysfunction involves the upregulation of the cholinergic receptor CHRNB2. Overall, these findings indicate that systems pharmacology could provide an alternative approach for exploring the complex MOAs of TCM and advance the comprehensive understanding of TCM.

## Data Availability Statement

All datasets generated for this study are included in the article/**Supplementary Material**.

## Ethics Statement

The animal study was reviewed and approved by Guangzhou University of Chinese Medicine Animal Ethics Committee.

## Author Contributions

SF and JF conceived and designed the experiments. YuL and DL conducted the experiments and wrote the manuscript. QWu, YaL, CC, HL, HH, YX and XL provided and analyzed some of the data. HK, QWa, JF, and SF reviewed and revised the manuscript.

## Funding

This work was supported by the National Natural Science Foundation of China (Nos. 81603318, 81673627, and 81904265), the Youth Scientific Research Training Project of GZUCM (No. 2019QNPY05), the Guangzhou Science Technology and Innovation Commission Technology Research Projects (No. 201805010005), and Key R & D and extension projects in Henan Province (No. 192102310166).

## Conflict of Interest

The authors declare that the research was conducted in the absence of any commercial or financial relationships that could be construed as a potential conflict of interest.

## Supplementary Material

Supplementary Datasheet S1 The detailed information of compounds of KXS and their corresponding targe.

Supplementary Datasheet S2 The detailed information of C-T network.
