# Corrigendum: Systems pharmacology approach to investigate the mechanism of Kai-Xin-San in Alzheimer’s disease

**DOI:** 10.3389/fphar.2023.1239060

**Published:** 2023-10-06

**Authors:** Yunxia Luo, Dongli Li, Yanfang Liao, Chuipu Cai, Qihui Wu, Hanzhong Ke, Xinning Liu, Huilin Li, Honghai Hong, Yumin Xu, Qi Wang, Jiansong Fang, Shuhuan Fang

**Affiliations:** ^1^ Science and Technology Innovation Center, Guangzhou University of Chinese Medicine, Guangzhou, China; ^2^ Department of Endocrinology, Fourth Clinical Medical College, Guangzhou University of Chinese Medicine, Shenzhen, China; ^3^ Department of Cancer Immunology and Virology, Dana-Farber Cancer Institute, Boston, MA, United States; ^4^ Department of Medicine, Harvard Medical School, Boston, MA, United States; ^5^ Department of Clinical Laboratory, The Third Affiliated Hospital of Guangzhou Medical University, Guangzhou, China; ^6^ Department of Encephalopathy First Affiliated Hospital of Henan University of Chinese Medicine, Zhengzhou, China; ^7^ Institute of Clinical Pharmacology, Guangzhou University of Chinese Medicine, Guangzhou, China

**Keywords:** systems pharmacology, Kai-Xin-San, Alzheimer’s disease, cholinergic system, neuroinflammation

In the published article, there was an error in Figure 8 as published. The Iba1 fluorescence image of the cortex and corresponding merged image in the KXS-L group was mistakenly displayed. The corrected Figure 8 and its caption appear below.

**FIGURE 8:**
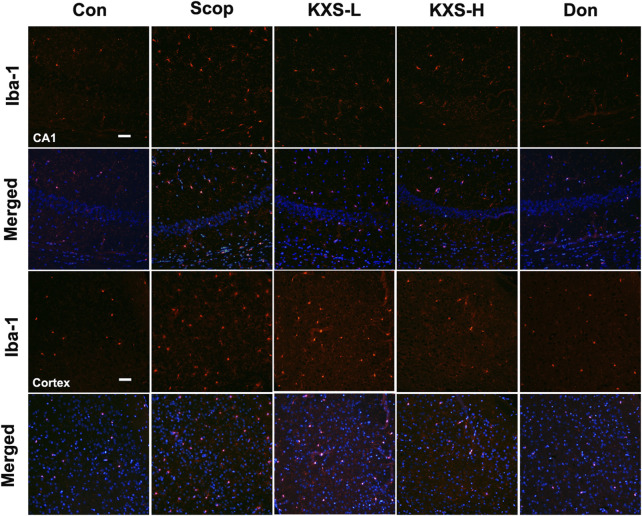
Kai-Xin-San (KXS) attenuates microglia activation in scopolamine (SCOP)-induced mice. Immunofluorescence analysis in the hippocampus (CA1) and cortex. Microglia were stained with anti-Iba-1 (red) and the nuclei were stained with DAPI (blue). Scale bar: 50 mm. Con, control group; SCOP, scopolamine; KXS-L, low-dose Kai-Xin San (1.4 g/kg); KSX-H, high-dose Kai-Xin San (2.8 g/kg); Don, donepezil.

The authors apologize for this error and state that this does not change the scientific conclusions of the article in any way. The original article has been updated.

