# Supplementary figures and images for: Systems Pharmacology Approach to Investigate the Mechanism of Kai-Xin-San in Alzheimer’s Disease
Source: Front Pharmacol. 2020 Apr 3;11:381. doi: 10.3389/fphar.2020.00381 (PMC7147119; doi:10.3389/fphar.2020.00381)

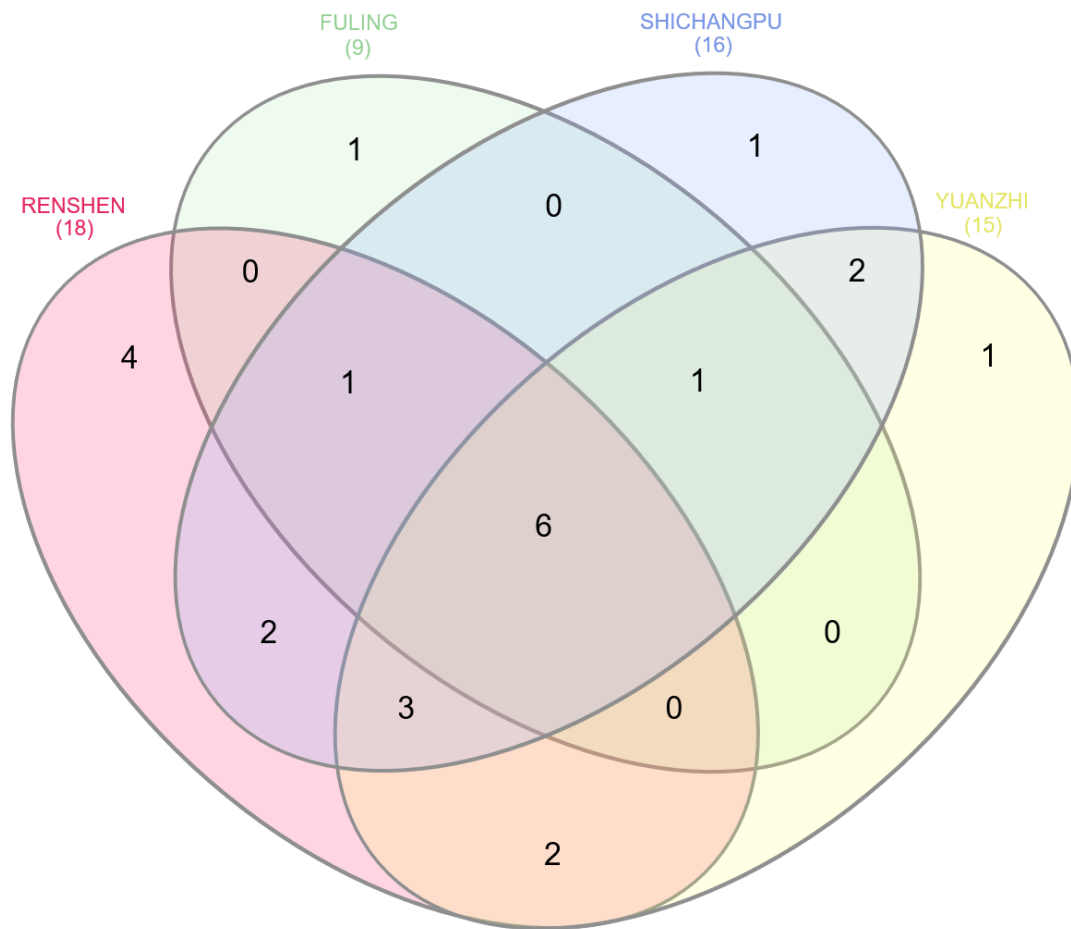

**FIGURE S1** | Venn diagram of 39AD-associated targets of KXS.

Supplement: Supplementary file 3 [file Image_1.pdf]
